# Comparative gut microbiota and resistome profiling of intensive care patients receiving selective digestive tract decontamination and healthy subjects

**DOI:** 10.1186/s40168-017-0309-z

**Published:** 2017-08-14

**Authors:** Elena Buelow, Teresita d. j. Bello González, Susana Fuentes, Wouter A. A. de Steenhuijsen Piters, Leo Lahti, Jumamurat R. Bayjanov, Eline A. M. Majoor, Johanna C. Braat, Maaike S. M. van Mourik, Evelien A. N. Oostdijk, Rob J. L. Willems, Marc J. M. Bonten, Mark W. J. van Passel, Hauke Smidt, Willem van Schaik

**Affiliations:** 1 Department of Medical Microbiology, University Medical Center Utrecht, Utrecht, The Netherlands; 2 Université Limoges, INSERM, CHU Limoges, UMR 1092, Limoges, France; 3 Laboratory of Microbiology, Wageningen University, Wageningen, The Netherlands; 4 Center for Immunology of Infectious Diseases and Vaccines, Bilthoven, The Netherlands; 5 Department of Pediatric Immunology and Infectious Diseases, The Wilhelmina Children’s Hospital, University Medical Center Utrecht, Utrecht, The Netherlands; 6 Department of Mathematics and Statistics, University of Turku, Turku, Finland; 7 Center of Infectious Disease Control, National Institute of Public Health and the Environment, Bilthoven, The Netherlands; 8 Institute of Microbiology and Infection, University of Birmingham, Edgbaston, Birmingham, B15 2TT United Kingdom

**Keywords:** Anti-bacterial agents, Antibiotic prophylaxis, Drug resistance, Microbial, Intensive care, Microbiome

## Abstract

**Background:**

The gut microbiota is a reservoir of opportunistic pathogens that can cause life-threatening infections in critically ill patients during their stay in an intensive care unit (ICU). To suppress gut colonization with opportunistic pathogens, a prophylactic antibiotic regimen, termed “selective decontamination of the digestive tract” (SDD), is used in some countries where it improves clinical outcome in ICU patients. Yet, the impact of ICU hospitalization and SDD on the gut microbiota remains largely unknown. Here, we characterize the composition of the gut microbiota and its antimicrobial resistance genes (“the resistome”) of ICU patients during SDD and of healthy subjects.

**Results:**

From ten patients that were acutely admitted to the ICU, 30 fecal samples were collected during ICU stay. Additionally, feces were collected from five of these patients after transfer to a medium-care ward and cessation of SDD. Feces from ten healthy subjects were collected twice, with a 1-year interval. Gut microbiota and resistome composition were determined using 16S rRNA gene phylogenetic profiling and nanolitre-scale quantitative PCRs.

The microbiota of the ICU patients differed from the microbiota of healthy subjects and was characterized by lower microbial diversity, decreased levels of *Escherichia coli* and of anaerobic Gram-positive, butyrate-producing bacteria of the *Clostridium* clusters IV and XIVa, and an increased abundance of Bacteroidetes and enterococci. Four resistance genes (*aac(6′)-Ii*, *ermC*, *qacA*, *tetQ*), providing resistance to aminoglycosides, macrolides, disinfectants, and tetracyclines, respectively, were significantly more abundant among ICU patients than in healthy subjects, while a chloramphenicol resistance gene (*catA*) and a tetracycline resistance gene (*tetW*) were more abundant in healthy subjects.

**Conclusions:**

The gut microbiota of SDD-treated ICU patients deviated strongly from the gut microbiota of healthy subjects. The negative effects on the resistome were limited to selection for four resistance genes. While it was not possible to disentangle the effects of SDD from confounding variables in the patient cohort, our data suggest that the risks associated with ICU hospitalization and SDD on selection for antibiotic resistance are limited. However, we found evidence indicating that recolonization of the gut by antibiotic-resistant bacteria may occur upon ICU discharge and cessation of SDD.

**Electronic supplementary material:**

The online version of this article (doi:10.1186/s40168-017-0309-z) contains supplementary material, which is available to authorized users.

## Background

The human gut microbiota comprises 10^13^–10^14^ bacterial cells that belong to hundreds of different species. The gut microbiota plays an important role in numerous metabolic, physiological, nutritional, and immunological processes of the human host [1]. In healthy individuals, the gut microbiota mostly consists of bacteria that have a commensal or mutualistic relationship with the human host. Critically ill patients, however, frequently have an extremely dysbiotic gut microbiota that is characterized by intestinal overgrowth with multi-drug resistant opportunistic pathogens of the phylum Proteobacteria (e.g., *Escherichia coli*) and the genus *Enterococcus*, while the abundance of commensal Bacteroidetes and Firmicutes is decreased [2–5]. The high levels of aerobic, opportunistic pathogens in the gut during critical illness are likely contributing to the burden of respiratory and bloodstream infections with these organisms in critically ill patients [6].

Selective digestive tract decontamination (SDD) aims to reduce the risk of nosocomial infections in ICU patients. SDD is used to eradicate opportunistic pathogens from the patients, while minimally impacting commensal bacteria [7]. In SDD, a paste containing the antibiotics colistin and tobramycin, and the antifungal amphotericin B, is applied to the oropharynx of ICU patients. The patients also receive a suspension of colistin, tobramycin, and amphotericin B via a nasogastric tube. These antimicrobials are applied from the day of ICU admission until ICU discharge. In addition, a third-generation cephalosporin (usually either cefotaxime or ceftriaxone) is administered intravenously during the first four days of ICU stay. SDD lowers patient mortality during ICU stay in settings with a low prevalence of antibiotic resistance and reduces the costs associated with ICU hospitalization [8, 9]. Selection of bacteria that are resistant to the antimicrobials used in SDD remains a major concern [10, 11], although this is not supported by the results of clinical trials in which conventional culture techniques were used to screen for antibiotic resistance among nosocomial pathogens [12].

The patient gut not only is a potential source for opportunistic pathogens but also forms a large reservoir for antibiotic resistance genes, termed the gut resistome [13–17]. The use of antibiotics may favor the selection for antimicrobial resistance genes (ARGs) among members of the gut microbiota, thus increasing the likelihood of horizontal spread of ARGs between commensals and opportunistic pathogens co-residing in the gut [16]. During the administration of SDD, the gut resistome of patients is monitored by the cultivation of resistant bacteria from rectal swabs or feces, as part of routine diagnostics. However, methods that rely on microbial culture capture only a fraction of the gut resistome, since anaerobic commensals, which are the main reservoir of ARGs in the gut microbiota, are difficult to culture [18–20]. Thus, culture-independent methods are needed to comprehensively assess the impact of antibiotic prophylaxis on the microbiota and resistome of ICU patients.

Here, we used the 16S ribosomal RNA (rRNA) gene-targeted Human Intestinal Tract Chip (HITChip) and nanolitre-scale quantitative PCR (qPCR) targeting 81 ARGs to determine the dynamics of gut microbiota composition and resistome in patients receiving SDD during ICU hospitalization. We contrast these findings in ICU patients with the composition of the microbiota and resistome of healthy subjects.

## Methods

### Study population

All included patients (*n* = 10) were acutely admitted to the ICU of the University Medical Center Utrecht from the community and had not been hospitalized in the previous six months, with the exception of patient 105 who was hospitalized for five days prior to transfer to the ICU and start of SDD. None of the patients were treated with antibiotics in six months prior to ICU hospitalization. All patients received SDD from the start of ICU stay until ICU discharge. SDD consists of 1000 mg of cefotaxime intravenously four times daily for four days; an oropharyngeal paste containing polymyxin E, tobramycin, and amphotericin B (each in a 2% concentration); and administration of a 10 mL suspension containing 100 mg polymyxin E, 80 mg tobramycin, and 500 mg amphotericin B via a nasogastric tube, four to eight times daily throughout ICU stay. All patients received additional antibiotics during ICU stay. Fecal samples of patients were collected by nursing staff upon defecation and stored at 4 °C for 30 min to 4 h, after which the samples were transferred to −80 °C. Seven patients included here (patient numbers 105, 108, 120, 163, 164, 165, and 169) were also included in a previous study where the dynamics of two aminoglycoside resistance genes in the gut microbiota of ICU patients was studied [20]. A total of 30 fecal samples were collected during ICU stay. Five additional fecal samples were collected after transfer to a medium-care ward and cessation of SDD. Additional file 1 includes detailed information on sampling time points and antibiotic usage of the ICU patients in this study.

Routine surveillance for colonization with aerobic Gram-negative bacteria in ICU patients was performed through culturing of rectal swabs on sheep blood agar and MacConkey agar. All suspected Gram-negative colonies were analyzed by Maldi-TOF for species identification. Antibiotic resistance phenotypes were determined using the Phoenix system (BD, Franklin Lakes, NJ, USA).

Fecal samples of healthy subjects were collected as part of the “Cohort study of intestinal microbiome among Irritable Bowel Syndrome patients and healthy individuals” (CO-MIC) study at two time points with a one-year interval between sampling. None of the individuals in this cohort received antibiotics. All included patients and healthy subjects were adults. Further demographic information on both cohorts is provided in Additional file 2. DNA from fecal samples of patients and healthy subject was isolated as previously described [21], using two rounds of bead beating and purification using the QIAamp DNA Stool Mini Kit columns (Qiagen; Venlo, The Netherlands).

### Gut microbiota profiling by HITChip

The HITChip is a validated phylogenetic array produced by Agilent Technologies (Palo Alto, CA) and developed at Wageningen University, The Netherlands [22, 23]. It contains over 4800 oligonucleotides targeting the V1 and the V6 region of the 16S rRNA gene from 1132 microbial phylotypes present in the human gut [22]. The full-length 16S rRNA gene was amplified from fecal DNA, and PCR products were further processed and hybridized to the microarrays as described previously [24]. Data analyses were performed using R (www.r-project.org), including the microbiome package (https://github.com/microbiome). Bacterial associations in the different patient groups and healthy subjects were assessed using principal component analysis (PCA) as implemented in CANOCO 5.0 [25]. Redundancy analysis (RDA) was performed as implemented in CANOCO 5.0 to determine the associations between microbiota composition (based on 130 genus-like groups included in the HITChip) and explanatory host-associated variables (age, gender, body mass index (BMI), and sample source, i.e., ICU patient or healthy subject). Significance of the explanatory variables was assessed by Monte Carlo permutation testing (MCPT). Statistical testing for the differences in microbiota composition between ICU patients and healthy subjects was performed by the non-parametric Mann-Whitney *U* test. All *P* values were corrected for false discovery rate (FDR) by the Benjamini and Hochberg method [26], and corrected *P* values (*q*) below 0.05 were considered significant.

### Quantification of *E. coli* by qPCR

qPCRs for the quantification of *E. coli* were performed with primers that were previously described [27], using serial dilutions of genomic DNA of *E. coli* DH5α to generate a standard curve. The quantification of 16S rRNA was performed with primers described in [28]. The PCR conditions were identical to the qPCR conditions for the detection of *mcr-1* (described below).

### qPCR analysis of antibiotic resistance genes

qPCR analysis was performed using the 96.96 BioMark™ Dynamic Array for Real-Time PCR (Fluidigm Corporation, San Francisco, CA), according to the manufacturer’s instructions, with the exception that the annealing step in the PCR was held at 56 °C. Fecal DNA was first subjected to 14 cycles of specific target amplification using a 0.18-μM mixture of all primer sets, excluding the 16S rRNA primer sets, in combination with the Taqman PreAmp Master Mix (Applied Biosystems), followed by a fivefold dilution prior to loading samples onto the Biomark array for qPCR. Thermal cycling and real-time imaging was performed on the BioMark instrument, and Ct values were extracted using the BioMark Real-Time PCR analysis software.

### Target selection, primer design, and primer validation

The primer set used in the qPCR assays covered 81 antimicrobial resistance genes (ARGs) of 14 resistance gene classes (Additional file 3). Primers were designed for the ARGs that are most commonly detected in the gut microbiota of healthy individuals [14, 15] and clinically relevant ARGs, including genes encoding extended spectrum β-lactamases (ESBLs), carbapenemases, and proteins involved in vancomycin resistance. Primer design was performed using Primer3 [29] with its standard settings with a product size of 80–120 bp and a primer melting temperature of 60 °C. The universal primers for 16S rRNA genes were previously described by Gloor et al. [28]. Forward and reverse primers were evaluated in silico for cross hybridization using BLAST [30] and were cross-referenced against ResFinder [31] to ensure the correct identity of the targeted genes. All primers that aligned with more than ten nucleotides at their 3′ end to another primer sequence were discarded and redesigned. Additionally, all primer sets were aligned to all resistance genes that were targeted in this PCR analysis to test for cross hybridization with genes other than the intended target resistance gene. Primers that aligned with more than ten nucleotides at their 3′ end sequence with a non-target resistance gene were discarded and redesigned. A reference sample consisting of pooled fecal DNA from different patients was loaded in a series of fourfold dilutions and was used for the calculation of primer efficiency. All primers whose efficiency was experimentally determined to be between 80 and 120% were used to determine the normalized abundance of the target genes. The detection limit on the Biomark system was set to a CT value of 20, as recommended by the manufacturer. In addition, to assess primer specificity, we performed melt curve analysis using the Fluidigm melting curve analysis software (http://fluidigm-melting-curve-analysis.software.informer.com/). All PCRs were performed in triplicate, and sample-primer combinations were only included in the analysis when all triplicate reactions resulted in a CT value below the detection limit.

After completion of the nanolitre-scale qPCR assays, the transferable colistin resistance gene *mcr-1* was described [32]. To detect and quantify *mcr-1*, we developed primers (qPCR-mcr1-F: 5′-TCGGACTCAAAAGGCGTGAT-3′ and qPCR-mcr1-R: 5′-GACATCGCGGCATTCGTTAT-3′) for use in a standard qPCR assay. The *mcr-1* gene was synthesized based on the sequence described in [32] by Integrated DNA Technologies (Leuven, Belgium) and used as a positive control in our assays. The qPCR was performed using Maxima SYBR Green/ROX qPCR Master Mix (Thermo Scientific, Leusden, The Netherlands) and a StepOnePlus instrument (Applied Biosystems, Nieuwekerk a/d IJssel, The Netherlands) with 5 ng DNA in the reaction and the following program: 95 °C for 10 min and subsequently 40 cycles of 95 °C for 15 s and 56 °C for 1 min.

### Calculation of normalized and cumulative abundance

Normalized abundance of resistance genes was calculated relative to the abundance of the 16S rRNA gene (CT_ARG_ − CT_16S rRNA_), resulting in a log2-transformed estimate of ARG abundance. Statistical analysis was performed using GraphPad Prism (La Jolla, CA). Visualization of the qPCR data in the form of a heat map was performed using Microsoft Excel. Statistical testing of the differences in the abundance of resistance genes in ICU patients versus healthy subjects was performed with the non-parametric Mann-Whitney *U* test with a false discovery rate (Benjamini Hochberg) <0.05 to correct for multiple testing.

## Results

### Microbiota dynamics in ICU patients and healthy subjects

**Fig. 1:**
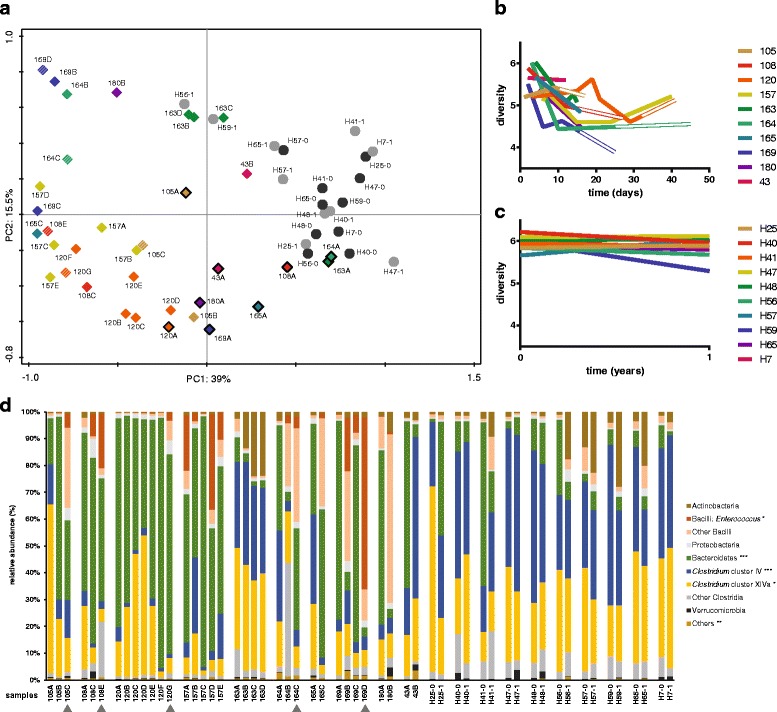
Dynamics of gut microbiota composition and diversity in ICU patients and healthy subjects. **a** Principal component analysis (PCA) of gut microbiota composition of ICU patients. *Dashed symbols* indicate fecal samples collected after ICU discharge and continued hospitalization in a medium-care ward. Fecal samples that were collected in the first 5 days of ICU hospitalization are indicated by a *black line* around the symbol. Fecal samples of healthy subjects were collected at two time points with 1-year interval, indicated with *black* and *gray circles*, respectively. **b** Diversity (Shannon index) of the microbiota of ICU patients. *Double lines* indicate hospitalization in a medium-care ward. **c** Diversity (Shannon index) of the microbiota of healthy subjects. **d** Gut microbiota composition of patients and healthy subjects. *Stacked bar charts* represent the abundance of different major taxa in the gut microbiota of ICU patients and healthy subjects. Among Bacilli, the genus *Enterococcus* has been highlighted, as SDD has previously been shown to select for colonization with enterococci [36, 37]. Fecal samples that were collected after ICU discharge and during medium-care hospitalization are indicated by *gray triangles*. Statistically significant differences of the abundance of taxa in the gut microbiota of patients during ICU hospitalization and healthy subjects are indicated in the legend (*q < 0.05; **q < 0.01; ***q < 0.001; Mann-Whitney *U* test with Benjamini-Hochberg correction for multiple testing)

Ten ICU patients and ten healthy subjects were included in this study. There were no statistically significant differences between the cohorts in their gender distribution and BMI. The age of the ICU patients was significantly (*P* < 0.05; Mann-Whitney *U* test) higher (median 57, interquartile range 49–74) than the age of the healthy subjects (median 42.5, interquartile range 31–51; Additional file 2). Global changes in the gut microbiota of healthy subjects and ICU patients were visualized by principal component analysis (Fig. 1a). The microbiota profiles of healthy subjects clustered together, indicating that they had stable and broadly comparable microbiota profiles, which were clearly distinct from the microbiota profiles of patients during and after ICU stay. These profiles covered a larger area in the PCA plot, indicating that the differences in the microbiota composition of patients were larger than that of healthy subjects. The composition of the gut microbiota in patient samples collected during and after ICU hospitalization was markedly altered, with samples clustering away from the healthy controls. RDA showed that both sample source (i.e., whether samples were taken from ICU patients or healthy subjects) and, to a lesser extent, BMI significantly explained microbiota composition variance (sample source: *q* = 0.007; BMI: *q* = 0.048, Additional file 4). The other tested variables (age and gender) did not significantly contribute to the variability in microbiota composition. Collectively, the tested variables explained 30% of microbiota composition variance.

The diversity of the microbiota, as quantified by Shannon’s diversity index, was significantly lower in ICU patients compared to healthy subjects (5.90 ± 0.20 vs 5.19 ± 0.46, respectively; *P* < 0.001, Student’s *t* test). The diversity of the microbiota of ICU patients was highly dynamic (Fig. 1b). Several patients (#108, #163, #164, #165, and #169) experienced a rapid loss of diversity in the first days of ICU stay. In contrast, the diversity of the microbiota was more stable in healthy subjects when comparing samples that were collected 1 year apart (Fig. 1c). Compared to healthy subjects, the microbiota of patients during ICU hospitalization was characterized by a significantly higher abundance in the taxa Bacteroidetes and Bacilli: *Enterococcus* and a lower abundance of the taxa *Clostridium* cluster IV and XIVa (Fig. 1d).

**Fig. 2:**
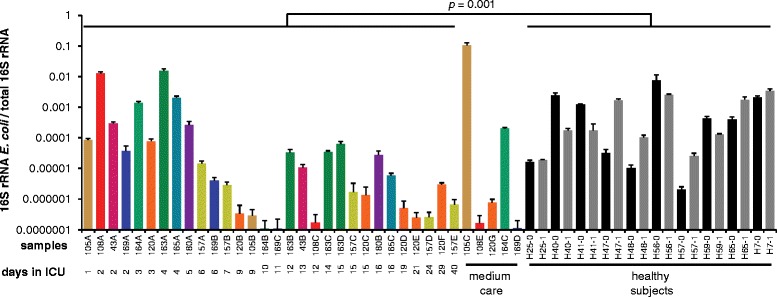
Abundance of *E. coli* in the gut microbiota of ICU patients and healthy subjects. Quantification of *E. coli* 16S rRNA gene copies relative to total 16S rRNA gene copies, performed by qPCR with three technical replicates. *Error bars* indicate standard deviation. Samples are ordered by time of sampling during ICU stay. The color coding of the samples is unique for each patient and is identical to Fig. 1. Statistical testing was performed with the Mann-Whitney *U* test

We performed quantitative PCRs to accurately determine the abundance of *E. coli*, one of the primary targets of SDD, in the gut microbiota of patients and healthy subjects (Fig. 2). The abundance of *E. coli* in samples of ICU patients was lower compared to the healthy subjects (*p* = 0.001; Mann-Whitney *U* test). Notably, upon ICU discharge, cessation of SDD, and transfer to a medium-care ward, the abundance of *E. coli* rebounded in one patient (#105) to levels surpassing those found in healthy individuals.

During ICU stay, routine surveillance by conventional microbiological culture was performed on all patients. *E. coli* could be cultured from six out of 73 rectal swabs that were collected during the patients’ ICU stay. Five *E. coli-*positive rectal swabs, of patients #43, #105, #108, #163, and #169, were collected within one day of ICU admission, while the sixth positive swab (of patient #165) was collected after 9 days of ICU stay. In addition, an *E. coli* strain from patient #105 with an ESBL-producing and tobramycin-resistant phenotype was isolated after ICU discharge, while the patient was in a medium-care ward. The *E. coli* strains isolated during ICU stay were susceptible to cephalosporins and aminoglycosides. All *E. coli* strains were susceptible to colistin.

### Resistome dynamics in ICU patients and healthy subjects

A total of 46 unique ARGs conferring resistance to 12 different classes of antimicrobials were detected in the DNA isolated from fecal samples of hospitalized patients and healthy subjects (Additional file 5). The number of detected resistance genes per sample ranged between 6 and 38. Eleven resistance genes were detected in >80% of healthy subjects and critically ill patients. This highly prevalent set of resistance genes included tetracycline resistance genes (*tetO*, *tetQ*, *tetM*, *tetW*), two aminoglycoside resistance genes (*aph(3′)-III* and an *aadE-*like gene), the bacteroidal β-lactam resistance gene *cblA*, and the macrolide resistance gene *ermB*.

Genes associated with major antibiotic resistance threats, including those identified by the Centers for Disease Control, were relatively rare. Genes encoding for extended-spectrum beta-lactamases (ESBLs) were not detected in healthy subjects. In two ICU patient samples (#105C and #108C), however, the ESBL genes *bla*_*CTX-M*_ and *bla*_*DHA*_, respectively, could be detected. Sample #105C was collected after ICU discharge and cessation of SDD, while sample #108C was collected after 12 days of ICU hospitalization and SDD treatment. The carbapenemase *bla*_*KPC*_ was detected in a single patient (patient #180), but only in the first sample (collected after five days in the ICU) and not in the second sample, which was collected after 16 days of ICU hospitalization. No other ESBL- or carbapenemase-producing strains were isolated from the patients during ICU hospitalization. Other enterobacterial β-lactamases were found to be widespread in our resistome analysis. The *bla*_*AMPC*_ β-lactamase was present in 37% of samples, with nine of ten patients and eight of ten healthy subjects having detectable levels of *bla*_*AMPC*_ at one or more sampling points. The *bla*_*TEM*_ β-lactamase was present in 26% of samples, corresponding with five of ten patients and four of ten healthy subjects in which this gene was detectable at one or more sampling points. None of the samples were positive for the carbapenemases *bla*_*NDM*_ and *bla*_*OXA*_ or the transferable colistin resistance gene *mcr-1.* Among resistance genes that are associated with Gram-positive pathogens, the staphylococcal methicillin resistance gene *mecA* was detected in 13 samples from eight of ten patients, but not in samples of healthy subjects. The vancomycin resistance gene *vanB* was present in five samples from three of ten patients and six samples from four of ten healthy subjects.

**Fig. 3:**
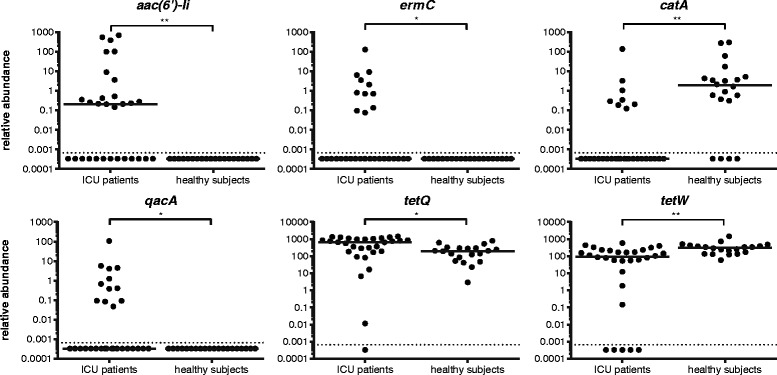
Antimicrobial resistance genes present at significantly higher or lower levels in the microbiota of ICU patients, compared to healthy subjects. ARGs that are present at significantly higher (*aac(6′)-Ii*, *ermC*, *qacA*, and *tetQ*) or lower (*catA* and *tetW*) abundance in ICU patients, compared to healthy subjects, are shown. Testing for statistically significant differences was performed by the Mann-Whitney *U* test, with Benjamini-Hochberg correction for multiple testing (*q < 0.05; **q < 0.01). The *horizontal line* denotes the median value. The detection limit of the qPCR assay is indicated with the *dashed line*

A comparison of the abundance of individual ARGs in samples that were collected during ICU stay, versus samples from healthy subjects, revealed that four ARGs (*aac(6′)-Ii*, *ermC*, *qacA*, *tetQ*) were significantly more abundant in ICU patients, while two ARGs (*catA* and *tetW*) were significantly more abundant in healthy individuals (Fig. 3).

## Discussion

Current guidelines in The Netherlands recommend topical antibiotic decontamination in ICU patients with an expected ICU stay of two days or longer. Yet, the original claim that these interventions do not affect harmless anaerobic intestinal bacteria [7] has recently been questioned [20, 33]. While culture-based studies did not demonstrate selection for antibiotic-resistant opportunistic pathogens during SDD treatment [9, 12, 34, 35], concerns remain that selection for antibiotic resistance genes occurs in the gut microbiota of patients that are treated by SDD during their stay in the ICU.

The current study describes the composition of the gut microbiota and the resistome of ICU patients receiving SDD during ICU stay and compares these findings to the microbiota and resistome of healthy subjects. We were not able to include an ICU control group that was not treated with SDD, as this would be a breach of clinical guidelines for ICU patients in our country. For this reason, it is not possible to disentangle the effects of SDD from other factors that affect the composition of the microbiota during ICU stay, including underlying critical illness, parenteral feeding, and curative antibiotic therapy.

The gut microbiota of ICU patients in this study was characterized by a low diversity, the increased abundance of enterococci, and lower abundance of anaerobic Gram-positive, butyrate-producing bacteria of the *Clostridium* clusters IV and XIVa. These findings are in line with previous studies reporting selection for Gram-positive cocci [12, 20, 36, 37] and depletion of *Faecalibacterium prausnitzii* in ICU patients receiving SDD [33]. In addition, we were able to demonstrate that the abundance of *E. coli* was significantly lower in ICU patients than in healthy individuals. The suppression of *E. coli* in the SDD-treated ICU patients starkly contrasts with other studies in critically ill patients not receiving SDD, in which *E. coli* is present at higher levels than in healthy individuals [2, 3, 5]. This observation further supports previous studies which found that SDD is successful in suppressing outgrowth of *E. coli* in the gut microbiota of ICU patients [8, 9], corresponding to the original aim of SDD [7].

Notably, levels of *E. coli* increased again after ICU discharge in two of patients, reaching levels in the gut similar to, or even surpassing, those in healthy individuals. These findings suggest that a rapid regrowth or recolonization of the intestinal tract by *E. coli*, and possibly other aerobic Gram-negative bacteria, occurs upon cessation of prophylactic antibiotic therapy. In one of the post-ICU samples, an ESBL-producing *E. coli* strain was isolated. Indeed, this was the only sample in which the ESBL *bla*_CTX-M_ gene was detected in our resistome analysis. In the only prospective evaluation on the post-ICU effects of SDD, the implementation of SDD was not associated with higher infection rates after ICU discharge [38], but further studies are warranted to better quantify the risks associated with recolonization of the gut by multi-drug resistant, opportunistic pathogens upon ICU discharge, and cessation of SDD.

The qPCR-based analysis of the resistome confirms previous metagenomic studies, in showing that tetracycline and aminoglycoside resistance genes and bacteroidal β-lactamases are widespread in the human intestinal microbiota [14, 15, 18, 20]. Four resistance genes (*aac(6′)-Ii*, *ermC*, *qacA*, *tetQ*) were significantly more abundant among ICU patients than in healthy subjects. The *aac(6′)-Ii* gene is a specific chromosomal marker for the nosocomial pathogen *Enterococcus faecium* and provides low-level resistance to aminoglycosides [39]. Its high abundance in ICU patients is in line with the increased levels of enterococci in the microbiota of the patients. The increased abundance of the macrolide resistance gene *ermC* may have been selected for by the use of low doses of the macrolide erythromycin, which was used as an agent to accelerate gastric emptying during ICU stay in six patients. The increased abundance of *tetQ* in the gut microbiota of ICU patients may reflect the higher abundance of Bacteroidetes in ICU patients versus healthy subjects, as *tetQ* is widely distributed on conjugative transposons in this phylum [40]. Finally, the *qacA* gene confers resistance to a number of disinfectants, including the biguanidine compound chlorhexidine and the quaternary ammonium compound benzalkonium chloride [41, 42]. Disinfectants are widely used in ICUs as cleaning and infection control agents [43], and their use could select for *qacA* in the gut microbiota of patients.

Two resistance genes (*catA* and *tetW*) were more abundant in healthy individuals than in ICU patients. There is currently little information on the distribution of the *catA* gene among bacteria associated with the human gut microbiota, but the gene was frequently found in human feces in a recent study set in low-income human habitats [44]. The tetracycline resistance gene *tetW* is present in Gram-positive anaerobic gut commensals [45]*.*

Although SDD improves survival of ICU patients, its use remains controversial due to the risk for selection of antibiotic resistance among bacteria that populate the patient gut. In this observational study, we found little evidence for the selection of high-risk resistance determinants, like ESBLs, carbapenemases, or vancomycin resistance genes, in SDD-treated patients during their stay in the ICU. The increased abundance of the resistance genes *aac(6′)-Ii*, *ermC*, *qacA*, and *tetQ* in SDD-treated ICU patients in our study could be interpreted as being of limited concern. The first three resistance genes contribute to resistance in enterococci, either to relatively low concentrations of antibiotics (*aac(6′)-Ii*) or to classes of antimicrobials that are of limited relevance for the treatment of enterococcal infections (*ermC* and *qacA*). The *tetQ* gene provides resistance to tetracyclines in Bacteroidetes, but this class of antibiotics is scarcely used for the treatment of anaerobic infections [46]. The selection for enterococci occurring in this patient cohort may be of concern in settings where vancomycin-resistant enterococci (VRE) are endemic. VRE is still infrequent in The Netherlands, and the *vanB* resistance gene that was detected in both patients and healthy subjects is most likely associated with Gram-positive anaerobes [47]. It remains to be determined whether SDD leads to selection for VRE in countries where these bacteria are more prevalent.

## Conclusions

Our data support the notion that in settings with low levels of circulating antibiotic resistance genes, ICU hospitalization and SDD treatment does not lead to the selection of antibiotic resistance genes with high clinical relevance. However, it is difficult to generalize these findings from a Dutch hospital to other countries, where resistant bacteria are more prevalent. Our study also illustrates the relative lack of data regarding recolonization of the gut upon ICU discharge and cessation of SDD. Future studies into the emergence and spread of antibiotic resistance genes in ICU patients can benefit from the qPCR platform used here as it enables the rapid detection and quantification of antibiotic resistance genes in the gut microbiota. The detection of high-risk antibiotic resistance genes (encoding, e.g., ESBLs, carbapenemases or vancomycin resistance proteins) in the resistome of patients may lead to the implementation of targeted antibiotic therapy or infection control measures to minimize the risk for selection and spread of these resistance genes.

## Additional files

Additional file 1: Patient details. The antibiotics used in treatment of patients during hospitalization and time points at which fecal samples were collected are indicated. SDD indicates the administration of topical components of SDD, i.e., a paste containing polymyxin E, torbramycin, and amphotericin B (each at 2%) applied to the oropharynx and the administration of a 10 mL suspension containing 100 mg polymyxin E, 80 mg tobramycin, and 500 mg amphothericin B via nasogastric tube. Black lines indicate hospitalization at the ICU, and blue lines indicate hospitalization at a medium-care ward. (PDF 309 kb)

Additional file 2: Demographic details of patients and healthy subjects. Gender, age, weight, and body mass index (BMI) are indicated. The statistical analysis of differences between the two cohorts was performed with the χ^2^ test (for gender) and Mann-Whitney *U* test (for age, weight, and BMI). ND: not determined. (XLSX 11 kb)

Additional file 3: Primers used in this study. Primers were developed to target the indicated ARGs. Primer sequences in bold indicate ARGs which were detected in ≥1 sample. (DOCX 39 kb)

Additional file 4: Redundancy analysis (RDA) of variance in microbiota composition. RDA was performed as implemented in CANOCO 5.0 [25] to determine the associations between microbiota composition and explanatory host-associated variables. Significance of the explanatory variables was assessed by MCPT. Statistical testing for the differences in microbiota composition between ICU patients and healthy subjects was performed by the Mann-Whitney *U* test with FDR correction by the Benjamini and Hochberg method. (XLSX 9 kb)

Additional file 5: Dynamics of the resistome in ICU patients and healthy subjects. ARGs are grouped and color-coded according to resistance gene families (B: bacitracin, C: chloramphenicol; M: macrolides; P, polymyxins; Qa: quaternary ammonium compounds, S: sulphonamides; Tet: tetracyclines; T: trimethoprim; V: vancomycin). Abundance (log2-transformed) is visualized relative to 16S rRNA. The time points at which samples were collected during hospitalization are indicated. Patients are color-coded as in Fig. 1. Samples indicated with a lighter color have been collected after cessation of SDD during medium-care hospitalization. Red and green boxes indicate antibiotic resistance genes that were significantly (*q* < 0.05, Mann-Whitney test with Benjamini-Hochberg correction) more or less abundant, respectively, in the gut microbiota of ICU patients compared to healthy subjects. (PDF 331 kb)
