# Supplementary figures and images for: Comparative gut microbiota and resistome profiling of intensive care patients receiving selective digestive tract decontamination and healthy subjects
Source: Microbiome. 2017 Aug 14;5:88. doi: 10.1186/s40168-017-0309-z (PMC5554972; doi:10.1186/s40168-017-0309-z)

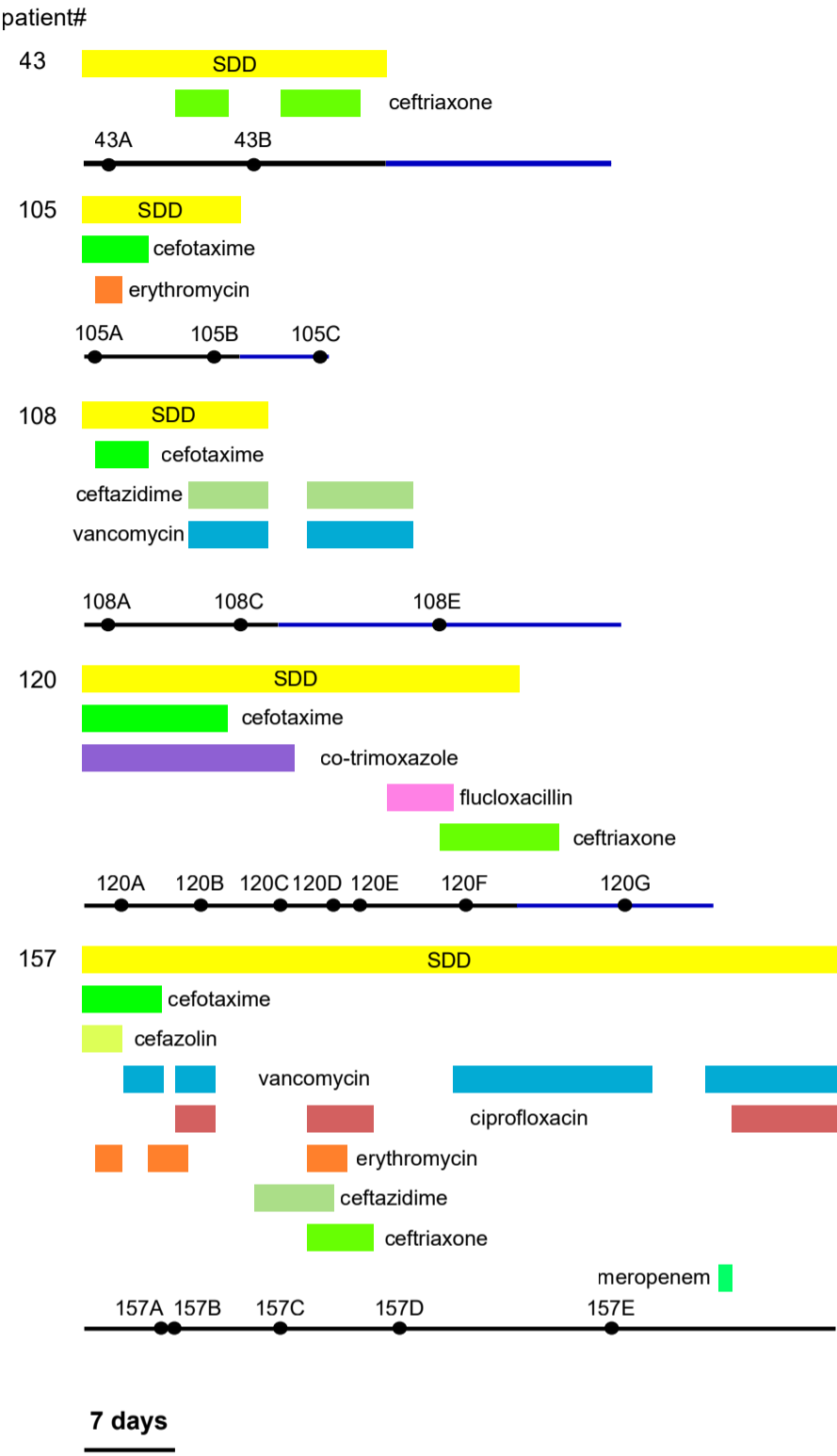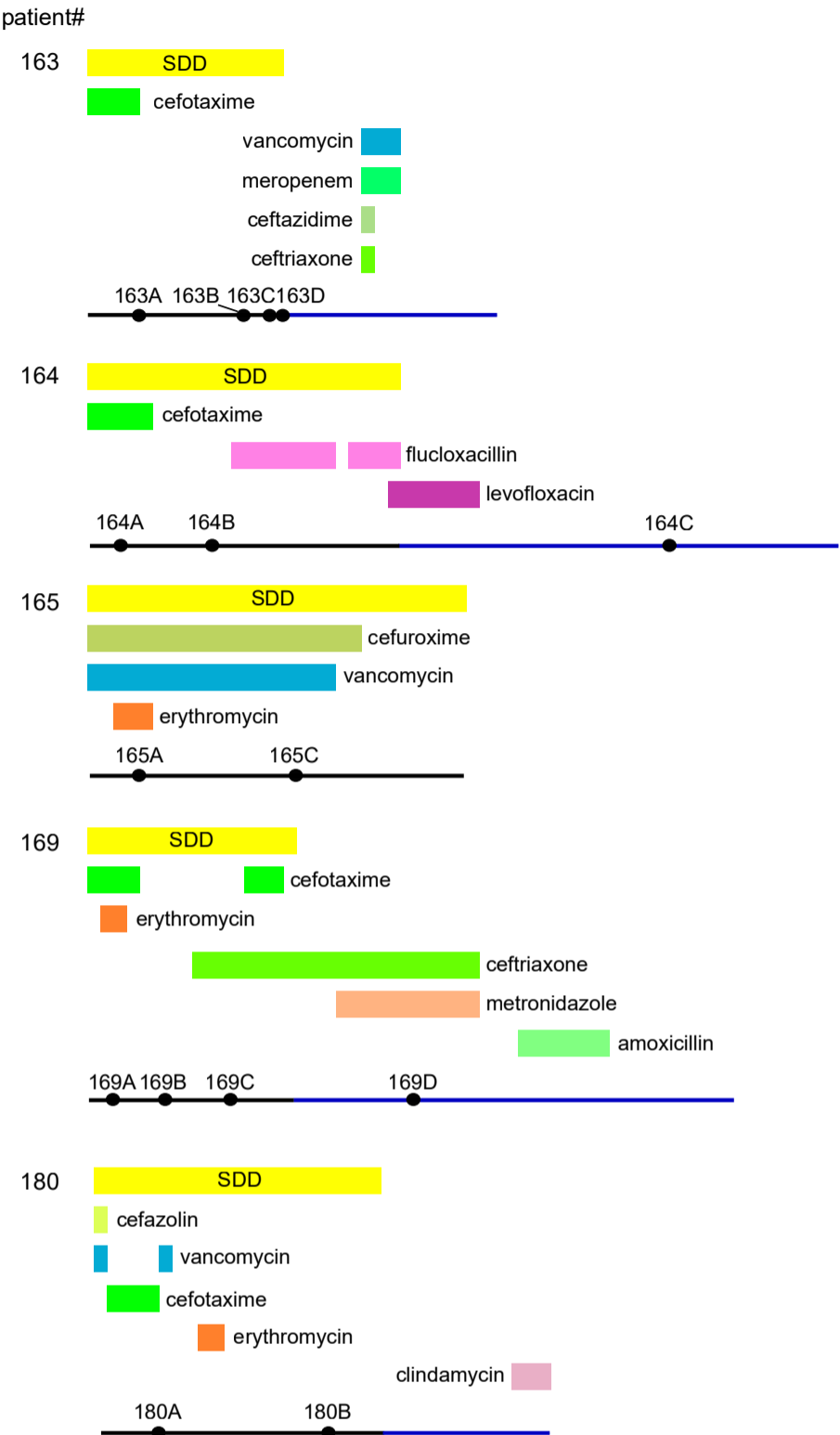

Supplement: Supplementary file 1 — Patient details. The antibiotics used in treatment of patients during hospitalization and time points at which fecal samples were collected are indicated. SDD indicates the administration of topical components of SDD, i.e., a paste containing polymyxin E, torbramycin, and amphotericin B (each at 2%) applied to the oropharynx and the administration of a 10 mL suspension containing 100 mg polymyxin E, 80 mg tobramycin, and 500 mg amphothericin B via nasogastric tube. Black lines indicate hospitalization at the ICU, and blue lines indicate hospitalization at a medium-care ward. (PDF 309 kb) [file 40168_2017_309_MOESM1_ESM.pdf]
