# Supplementary material for: Comparative gut microbiota and resistome profiling of intensive care patients receiving selective digestive tract decontamination and healthy subjects
Source: Microbiome. 2017 Aug 14;5:88. doi: 10.1186/s40168-017-0309-z (PMC5554972; doi:10.1186/s40168-017-0309-z)
Supplement: Supplementary file 3 — Primers used in this study. Primers were developed to target the indicated ARGs. Primer sequences in bold indicate ARGs which were detected in ≥1 sample. (DOCX 39 kb) [file 40168_2017_309_MOESM3_ESM.docx]

**Additional file 3. Primers used in this study.** Primers were developed to target targeted the indicated ARGs. Primer sequences in bold indicate ARGs which were detected in ≥1 sample.

| **Antimicrobial resistance gene** | **Accession number** | **Forward primer** | **Reverse primer** |
| --- | --- | --- | --- |
| *acrB* | YP_002396537.1 | CACGGTGACACAGGTTATCG | AAGGTCAGGGTGATCTGCAC |
| ***acrF*** | **CAR04877.2** | **ACTGACACCGGTTGATGTGA** | **GAGCAATAATCGAGGCGTTC** |
| ***tolC*** | **BAG78840.1** | **CTGAAAGAAGCCGAAAAACG** | **CGTCGGTAAGTGACCATCCT** |
| ***acrA*** | **ACI36997.1** | **GAAGGTAGCGACATCGAAGC** | **CTTTCGCCAGATCACCTTTC** |
| ***aph(3’)-III*** | **ACB90577.1** | **CCGGTATAAAGGGACCACCT** | **CTTTGGAACAGGCAGCTTTC** |
| ***aph(2”)-Ib*** | **AF207840.1** | **ATCAAATCCCTGCGGTAGTG** | **CAAGGGCATCCTTTTCCTTT** |
| ***aadE-*like gene** | **AAW34138.1** | **GCATGATTTCCTGGCTGATT** | **CCACAATTCCTCTGGGACAT** |
| *aac(6’)-aph(2”)* | ABY79711.1 | TCCAAGAGCAATAAGGGCATA | TGCCCTCGTGTAATTCATGT |
| ***aac(6')-Ii*** | **WP_002293989.1** | **AGACAGCTCGGCAGAAGAAG** | **ACCGTATTGAGGGATTGCAC** |
| ***aac(3’)-Ii(acde)*** | **HQ246166.1** | **TGACGTATGAGATGCCGATG** | **GAGAATGCCGTTTGAATCGT** |
| ***aac(6’)-Ib*** | **KM387722.1** | **TTGCAATGCTGAATGGAGAG** | **TGGTCTATTCCGCGTACTCC** |
| *aadA* | ADW23165.1 | GAACATAGCGTTGCCTTGGT | GCTGCGAGTTCCATAGCTTC |
| *aac(6')-IIa* | ACR24243.1 | GAACACTACCTGCCCAGAGC | GCGACGTACGACTGAGCATA |
| ***aph(2”)-I(de)*** | **AAC14693.1** | **CGGAGGTGGTTTTTACAGGA** | **TTGCTTCGGCAGATTATTGA** |
| *aph(3’)-Ia, -Ic* | CAQ58482.1 | ATTCTCACCGGATTCAGTCG | ATTCCGACTCGTCCAACATC |
| ***strB*** | **CAJ77026.1** | **GGCGATTATAGCCGATCAAA** | **CGCGACTGGAGAACATGATA** |
| ***bacA_2*** | **ABR38862.1** | **GAGGCATTGATCCTTGGTGT** | **AAACAATGCCGAACCGATAG** |
| ***bacA_1*** | **CAH05846.1** | **GGCTGCGTTACTGTCGTTTT** | **GGCCAATGATAAATGCATCC** |
| *bacA* | ACL18936.1 | AACTTCCCGTTCTGGTGCTA | CATAACGGGGATAGCGAGAA |
| *bla_GES_* | ABG47465.1 | CTGCTGCAATGACGCAGTAT | TATCTCTGAGGTCGCCAGGT |
| *bla_IMP_* | AJ640197.1 | GCTACCGCAGCAGAGTCTTT | CCCACCCGTTAACTTCTTCA |
| *bla_VIM_* | AM183120.1 | TGTCCGTGATGGTGATGAGT | TTTCAATCTCCGCGAGAAGT |
| *bla_ACC_* | AJ870923.1 | TTGTTACGCTACGTGCAAGC | CGATTTGAAATAGCCGGTGT |
| *bla_DHA_* | AHN96243.1 | AAAGTGCGCAAAGCCAGTAT | AAGATTCCGCATCAAGCTGT |
| *bla_IMI_* | U50278.1 | AGTCGATCCCAGCAGCTTTA | CCAAGAAACTGTGCATTCCA |
| *bla_CMY-1/MOX_* | AF357598.1 | GATCTGCTGCGTTTTGTGAA | CTACCGAGTAATGCCCTTGG |
| ***bla_AMPC_*** | **ABF06289.1** | **ACCGCTAAACAGTGGAATGG** | **GCAAGTCGCTTGAGGATTTC** |
| ***cepA*** | **CR626927.1** | **ATGTCCTGCCCTGGTAGTTG** | **CTTGCCCGTCGATAATGACT** |
| ***cepA_2*** | **AE016945.1** | **TGCACCAAGACGAAAGTCTG** | **ACAGTGCTTCTTTGCGGAAT** |
| *bla_BIC-1_* | GQ260093.1 | CCATCAGCGCACAACATAGT | CCAGAACGTTTTCCAGAAGC |
| ***cblA*** | **AAA66962.1** | **TGCCTGCGACATCTTGATAG** | **CCGTCTTCTGTTTCCGAGAG** |
| ***cfxA*** | **AY769933.1** | **GCGCAAATCCTCCTTTAACA** | **ACAATAACCGCCACACCAAT** |
| *bla_CMY-2_* | AAZ99133.1 | CGATCCGGTCACGAAATACT | CCTGCCGTATAGGTGGCTAA |
| ***bla_CTX-M_*** | **ABG46354.1** | **ACTATGGCACCACCAACGAT** | **GGTTGAGGCTGGGTGAAGTA** |
| ***bla_TEM_*** | **NP_775035.1** | **AAGCCATACCAAACGACGAG** | **TTGCCGGGAAGCTAGAGTAA** |
| *bla_SHV_* | AAV83796.1 | CTTTCCCATGATGAGCACCT | AGATCCTGCTGGCGATAGTG |
| *bla_NDM_* | CAZ39946.1 | ATATCACCGTTGGGATCGAC | TAGTGCTCAGTGTCGGCATC |
| *bla_OXA_* | AAP70012.1 | GTGGCATCGATTATCGGAAT | AGAGCACAACTACGCCCTGT |
| ***bla_KPC_*** | **AEL12451.1** | **TGGCTAAAGGGAAACACGAC** | **TAGTCATTTGCCGTGCCATA** |
| ***cat*** | **ABO92401.1** | **CAATCCTCAATCGACACGAA** | **GATTGTGTAGCAAGGCAGCA** |
| ***mdtL*** | **CAR15381.2** | **CGGACAAACCACGAGAAAAT** | **GAAGGTGAGGATCACCGAAA** |
| ***mdtF*** | **KEL93478.1** | **GGACCCGCAAAAACTCAATA** | **AGTTGACCACCGGAAATCTG** |
| *ermF* | BAD66041.1 | AGCACCCGCTTTTTCCTTAT | GATCAAGAGGGGCTTTAGGG |
| ***ermB*** | **BAH18720.1** | **GGTTGCTCTTGCACACTCAA** | **CTGTGGTATGGCGGGTAAGT** |
| *ermG* | 122586.NMB0066 | TGCTGTCTTTTACAGGCCACT | GCATATGTTCCAGTCCCTTCA |
| ***ermC*** | **BAE05991.1** | **TGAAATCGGCTCAGGAAAAG** | **GGTCTATTTCAATGGCAGTTACG** |
| ***mefA_10*** | **583346.CKR_2320** | **CCTGCAAATGGCGATTATTT** | **CCAAAGACCGCATAGGGTAA** |
| ***mefA_3*** | **286636.M6_SPY1166** | **TTACCCTATGCGGTCTTTGG** | **GAACCAGCTGCTGCGATAAT** |
| *macB* | ACR63203.1 | GGCTGGAAGACCGTACAGAG | GTTGGTTCATCGGCAAGAAT |
| *fosB* | NP_372857.1 | TTGAGCTTGCAGGCCTATG | GCCAATATTTAAATTCGCTGTCA |
| *cfr* | YP_003896025.1 | CAAACGAAGGGCAGGTAGAA | GACCACAAGCAGCGTCAATA |
| ***mfsA*** | **WP_002584949.1** | **AATATGCTCTCCGGGCTTTT** | **TTTGCACACCGTAAAATGGA** |
| *ermA* | AB047088.2 | GAGGGGTTTACCGCTTCTTT | ATCGGATCAGGAAAAGGACA |
| ***mecA*** | **YP_184944.1** | **TCCAGGAATGCAGAAAGACC** | **GGCCAATTCCACATTGTTTC** |
| ***arnA*** | **CAR03684.2** | **GAAATTCACCGTCTGGTCGT** | **GTGGTGCAACAGAAATCACG** |
| ***mdtO*** | **BAI33519.1** | **TTGTTGGCCTCTATCCAACC** | **TTAAGCGCTTGATGCATTTG** |
| ***qacA*** | **YP_536864** | **GACCCTTCTGGTACCCAACA** | **TCCCCATTTATCAGCAAAGG** |
| *qacC* | CAA86016.1 | TGGGCGGGACTAGGTTTAG | ACGAAACTACGCCGACTATGA |
| *acrP* | AKL33057.1 | CAGGCACTCCTTTCAGCTTC | GAGGCCGTGTTCAATTTGTT |
| *chvD* | CDX10534.1 | ATTCTGTGGCTGGAGCAGTT | GATCCACTTCGCAGATCCAT |
| ***qacE*** | **NC_001735.4** | **TCGGTGTTGCTTATGCAGTC** | **ATCAAGCTTTTGCCCATGAA** |
| *qnrA* | ACA43024.1 | ATTTCTCACGCCAGGATTTG | ACTGCAATCCTCGAAACTGG |
| *qnrB* | AFD54601.1 | CGATCTGACCAATTCGGAGT | ACGATGCCTGGTAGTTGTCC |
| *qnrC* | ACK75961.1 | GCAGAATTCAGGGGTGTGAT | AACTGCTCCAAAAGCTGCTC |
| *qnrS* | AEG74318.1 | TGGAAACCTACCGTCACACA | AATCGCATCGGATAAAGGTG |
| ***spc*** | **AAL05551.1** | **TGACGAACGCAATGTGATTT** | **TCAGCTGCCAGATCTTTTGA** |
| *vatA* | AAF24087.1 | AACAGCTTCTGCAGCAATGA | CCTTGAAAGGGGACATTGAA |
| ***vatB*** | **AAA86871.1** | **TGGGAAAAAGCAACTCCATC** | **TTCTGACCAATCCACACATCA** |
| *aadE* | CAZ55809.1 | TGTGCCGCAAAGAGATACTG | TTATCCCAACCTTCCACGAC |
| ***sul1*** | **ADB23338.1** | **AGGCTGGTGGTTATGCACTC** | **AAGAACCGCACAATCTCGTC** |
| ***tetQ*** | **Y08615.1** | **GCAAAGGAAGGCATACAAGC** | **AAACGCTCCAAATTCACACC** |
| *tetX* | ABQ05845.1 | CGGTACGCTGGATTTACACA | CATCGGAATTGCCTTTTTGT |
| ***tetW*** | **ACD97480.1** | **GGTGCAGTTGGAGGTTGTTT** | **AAATGACGGAGGGTTCCTTT** |
| ***tetM*** | **ACO22036.1** | **TTGATGCGGGAAAAACTACC** | **TACCTCTGTCCACGCTTCCT** |
| ***tetO*** | **EAQ71799.1** | **GCGTCAAAGGGGAATCACTA** | **CGGTATACTTCCGCCAAAAA** |
| ***tetB*** | **AAL09908.1** | **CAAAACTTGCCCCTAACCAA** | **GCTTTCAGGGATCACAGGAG** |
| *dfrA* | BAF39170.1 | AGCACGATAGTAGCCGCAGT | AAGGTTTTGGGGAAATCGTC |
| ***dfrF*** | **AEBU01000146.1** | **GATTGTTGCGAGGTCAAAGAA** | **CGCCCCATAATAACCACATT** |
| *vanUG* | ACR77286.1 | ATTTGCGAAACTCGGAAAAA | ACACCTCATTTTCGGGTACG |
| *vanR* | CAJ68489.1 | TGAAGCTGTATGGGGAGAAAA | TTTCGGGTTTTTAGAAGGTTCA |
| *vanA* | ACP19236.1 | GTGCGGTATTGGGAAACAGT | TGCGTTTTCAGAGCCTTTTT |
| ***vanB*** | **WP_032489746.1** | **CCTGCCTGGTTTTACATCGT** | **GCTGTCAATCAGTGCAGGAA** |
| *vanX* | NP_878017.1 | CCGGTTGACGGTTATGAAGT | CAGCCAGTTCTTTTGCCTTC |
| *cfr_2* | AJ249217.1 | GCCGGAGCTTTTCCTCTACT | GGTGCCGAAAGTCAAAACAT |
| **16S rRNA** | Gloor *et al.*, 2010 | **CAACGCGARGAACCTTACC** | **ACAACACGAGCTGACGAC** |
|  | PLoS ONE 5:e15406 |  |  |
|  |  |  |  |
