# Supplementary material for: Comparative gut microbiota and resistome profiling of intensive care patients receiving selective digestive tract decontamination and healthy subjects
Source: Microbiome. 2017 Aug 14;5:88. doi: 10.1186/s40168-017-0309-z (PMC5554972; doi:10.1186/s40168-017-0309-z)
Supplement: Supplementary file 5 — Dynamics of the resistome in ICU patients and healthy subjects. ARGs are grouped and color-coded according to resistance gene families (B: bacitracin, C: chloramphenicol; M: macrolides; P, polymyxins; Qa: quaternary ammonium compounds, S: sulphonamides; Tet: tetracyclines; T: trimethoprim; V: vancomycin). Abundance (log2-transformed) is visualized relative to 16S rRNA. The time points at which samples were collected during hospitalization are indicated. Patients are color-coded as in Fig. 1. Samples indicated with a lighter color have been collected after cessation of SDD during medium-care hospitalization. Red and green boxes indicate antibiotic resistance genes that were significantly (q < 0.05, Mann-Whitney test with Benjamini-Hochberg correction) more or less abundant, respectively, in the gut microbiota of ICU patients compared to healthy subjects. (PDF 331 kb) [file 40168_2017_309_MOESM5_ESM.pdf]

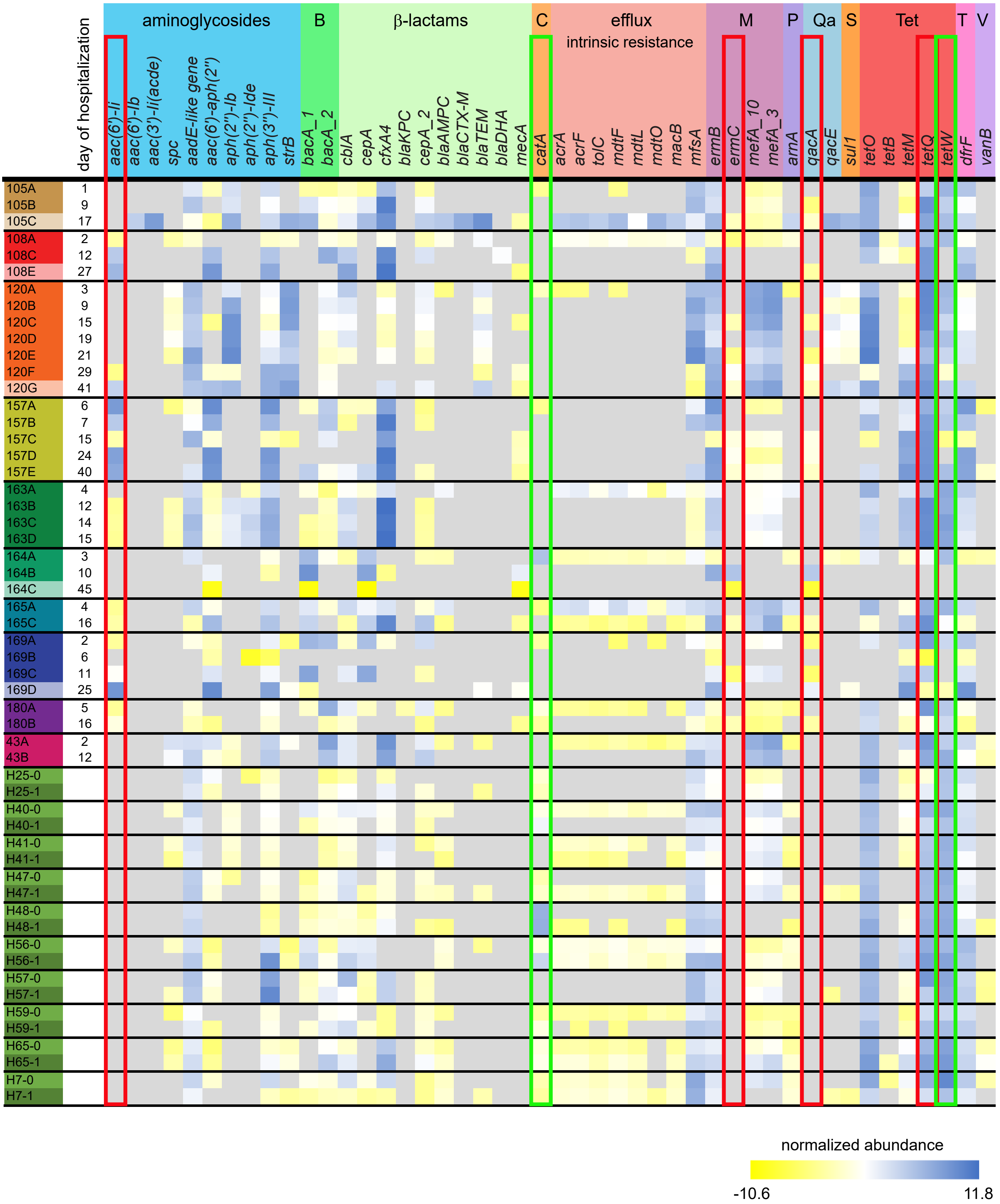

day of hospitalization
aminoglycosides
B
β-lactams
C
efflux
intrinsic resistance
M
P
Qa
S
Tet
T
V
aac(6')-Ii
aac(6')-Ib
aac(3')-Ii(acde)
spc
aadE-like gene
aac(6')-aph(2'')
aph(2'')-Ib
aph(2'')-Ide
aph(3'')-III
strB
bacA_1
bacA_2
cblA
cepA
cfxA4
blaKPC
cepA_2
blaAMPC
blaCTX-M
blaTEM
blaDHA
mecA
catA
acrA
acrF
tolC
mdtF
mdtL
mdtO
macB
mfsA
ermB
ermC
mefA_10
mefA_3
arnA
qacA
qacE
sul1
tetO
tetB
tetM
tetQ
tetW
dfrF
vanB
105A 1
105B 9
105C 17
108A 2
108C 12
108E 27
120A 3
120B 9
120C 15
120D 19
120E 21
120F 29
120G 41
157A 6
157B 7
157C 15
157D 24
157E 40
163A 4
163B 12
163C 14
163D 15
164A 3
164B 10
164C 45
165A 4
165C 16
169A 2
169B 6
169C 11
169D 25
180A 5
180B 16
43A 2
43B 12
H25-0
H25-1
H40-0
H40-1
H41-0
H41-1
H47-0
H47-1
H48-0
H48-1
H56-0
H56-1
H57-0
H57-1
H59-0
H59-1
H65-0
H65-1
H7-0
H7-1
normalized abundance
-10.6
11.8
